# Computed tomography colonography versus colonoscopy for detection of colorectal cancer: a diagnostic performance study

**DOI:** 10.1186/s12880-020-00446-7

**Published:** 2020-05-18

**Authors:** Junping sha, Jun chen, Xuguang lv, Shaoxin liu, Ruihong chen, Zhibing zhang

**Affiliations:** 1 Department of Radiology, Xiantao First People’s Hospital Affiliated to Yangtze University, Xiantao, 433000 Hubei China; 2 Department of Radiology, Renmin Hospital of Wuhan University, Wuhan, 430060 Hubei China; 3 Department of Gastroenterology, Xiantao First People’s Hospital Affiliated to Yangtze University, Xiantao, 433000 Hubei China

**Keywords:** Colonoscopy, Colorectal cancer, Computed tomographic colonography, Surgical pathology, Suspicious polyps

## Abstract

**Background:**

Colonoscopy is the reference standard for the detection of colorectal cancer but it is an invasive technique and has the risk of bowel perforation and bleeding. Unlike colonoscopy, sedation is not required in computed tomography colonography and requires additional reassurance endoscopy. The objectives of the study were to compare the diagnostic performance of computed tomography colonography against colonoscopy for a diagnosis of colorectal cancer.

**Methods:**

Data regarding any polyp ≥10 mm diameter (ø) and < 10 mm ø but suspicious polyps of computed tomography colonography (*n* = 318), colonoscopy (*n* = 318), and surgical pathology (*n* = 77) for symptomatic colorectal cancer patients were collected and analyzed. Lesion ulceration, extramural invasion, and/ or lesion shouldering was considered as a suspicious polyp. Beneficial scores for decision making of curative surgeries were evaluated for each modality. The cost of diagnosis of colorectal cancer was also evaluated.

**Results:**

Either of diagnosis showed polyps ≥10 mm ø in 27 patients and polyps of 50 patients were < 10 mm ø but suspicious. Therefore, a total of 77 patients were subjected to surgery. With respect to surgical pathology, sensitivities for computed tomographic colonography and colonoscopy were 0.961 and 0.831. For detection of ≥10 mm ø polyp, benefit score for computed tomographic colonography and colonoscopy were 0–0.906 diagnostic confidence and 0.035–0.5 diagnostic confidence. For polyps, ≥ 10 mm ø but not too many large polyps, colonoscopy had the risk of underdiagnosis. For < 10 mm ø but suspicious polyps, < 0.6 mm ø and < 2.2 mm ⌀ polyps could not be detected by computed tomographic colonography and colonoscopy, respectively. The computed tomographic colonography had less cost than colonoscopy (1345 ± 135 ¥/ patient vs. 1715 ± 241 ¥/ patient, *p* < 0.0001) for diagnosis of colorectal cancer.

**Conclusion:**

Computed tomographic colonography would be a non-inferior alternative than colonoscopy for a diagnosis of colorectal cancer.

**Level of evidence:**

III.

## Background

Colorectal cancer is the fourth most common cancer in China [1]. High red and processed meat intake in the Chinese population is responsible for colorectal cancer [2]. Several methods are available for the diagnosis of colorectal cancer but it is usually made by colonoscopy [3]. Colonoscopy is the reference standard for the detection of colorectal cancer [4] but it is an invasive technique and has the risk of bowel perforation and bleeding [5]. Sedation is also required which increases the risk especially in old aged patients [6]. Sedation is not required in computed tomography colonography [7]. Computed tomography colonography has a high sensitivity for large polyps in asymptomatic [8] and symptomatic [9] population but it images outside the organ [10], requires additional reassurance endoscopy to biopsy lead to increases the cost and burden of the diagnosis [11], and requires quality assurance for radiologists [12]. However, the study suggested that computed tomography colonography has the same sensitivity to colonoscopy for the detection of colorectal cancer [4] but has an issue of the small effect and the large variability in the study which may have type-II errors [13]. Large benign colorectal precursor mass lesions of invasive malignant cancers present challenges in both computed tomography colonography and colonoscopy [8]. In short, detection of colorectal cancer is critical irrespective of symptoms and age.

The objectives of the retrospective study were to compare diagnostic performance, cost, and safety of computed tomography colonography against colonoscopy for the diagnosis of colorectal cancer considering surgical pathology as a reference standard in symptomatic patients.

## Methods

### Study population

**Fig. 1:**
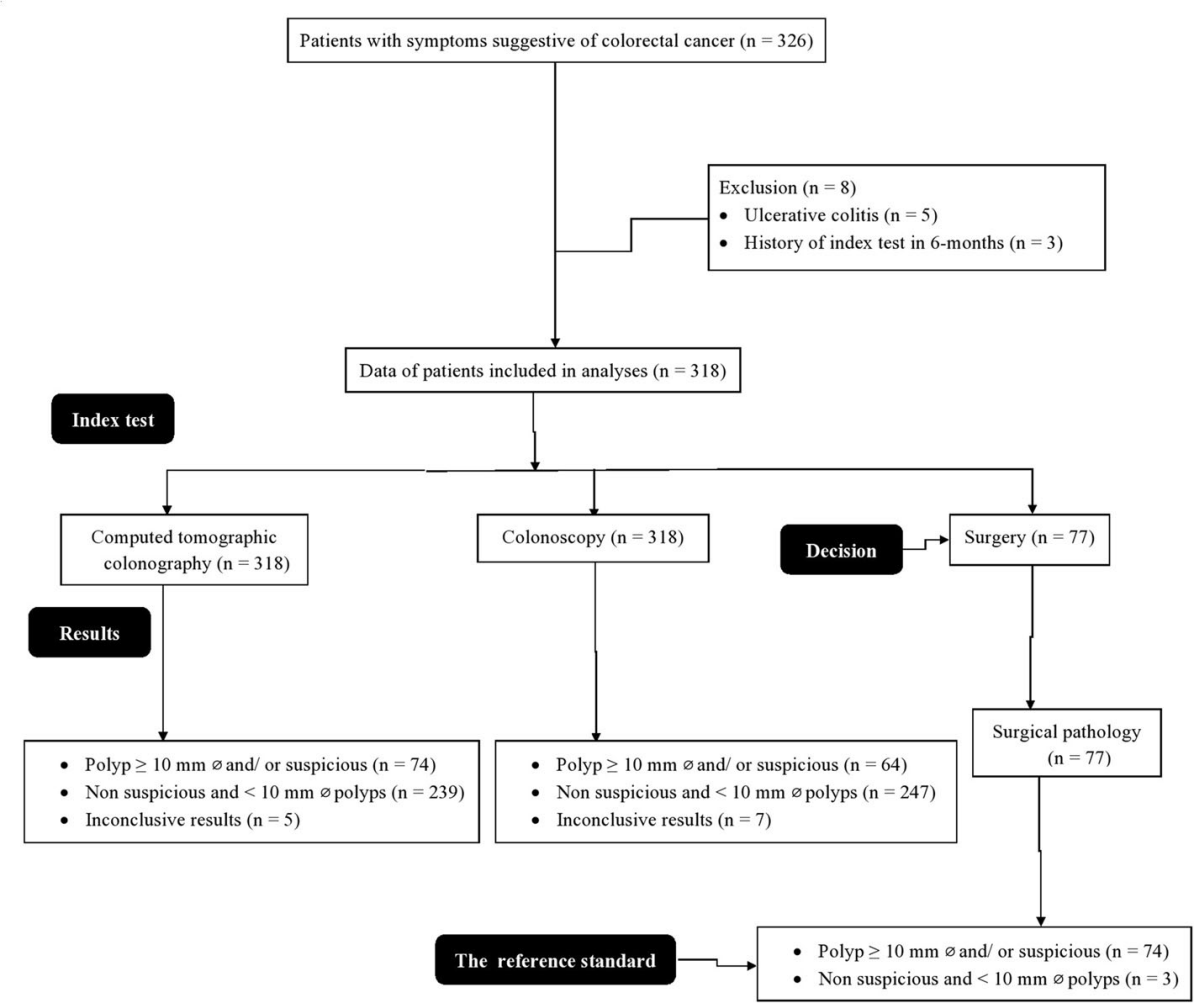
Study flow chart. ø: Diameter

From 15 January 2019 to 1 November 2019, a total of 326 patients were available at the department of gastroenterology of the Xiantao First People’s Hospital Affiliated to Yangtze University, Xiantao, Hubei, China and the Renmin Hospital of Wuhan University, Wuhan, Hubei, China who had symptom(s) suggestive of colorectal cancer such as abdominal pain, rectal bleeding, and/ or change in bowel habits, need whole-colon examinations, and performed both computed tomography colonography and colonoscopy. Among them, five patients have known diagnosis of ulcerative colitis and three patients were undergone whole-colon examinations in the last 6-months. Therefore, the data of these patients were not included in the analyses and data of 318 symptomatic patients suggestive of colorectal cancer were included in the analysis (Fig. 1).

### Computed tomographic colonography

All patients have injected 20 mg intravenous hyoscine (Buscopan, Sanofi, Berkshire, UK) before the examination. Intravenous contrast was administered on demand and computed tomographic colonography was performed as per international guidelines for good clinical practice under a single breath-hold of the patient using a multidetector instrument (GE Healthcare, New York, USA). Patients were scanned both supine and prone positions by radiologists (minimum of 3-years of experience in abdominal imaging) of institutes. Images acquired using 0.125 cm collimation, 0.1 cm reconstruction interval, 119 kVp, and 51–74 mAs fixed tube current-time product or 28–290 mA tube current modulation. The examinations involved identification of colorectal neoplasia (any polyp 10 mm or more in diameter (ø) and polyps less than 10 mm ø but suspicious).

### Colonoscopy

It was performed using video endoscopes (RetroView™ Colonoscopes EC34-i10T, PENTAX Medical, New Jersey, USA) after bowel preparation under midazolam/ fentanyl sedation. Gastroenterologists or colorectal surgeons (minimum of 3-years of experience) of institutes performed the colonoscopy. The examinations were performed as per the institutional protocol and detected lesions were evaluated by biopsies (performed by pathologists, a minimum 3-years of experience of institutes).

### Image analysis

**Fig. 2:**
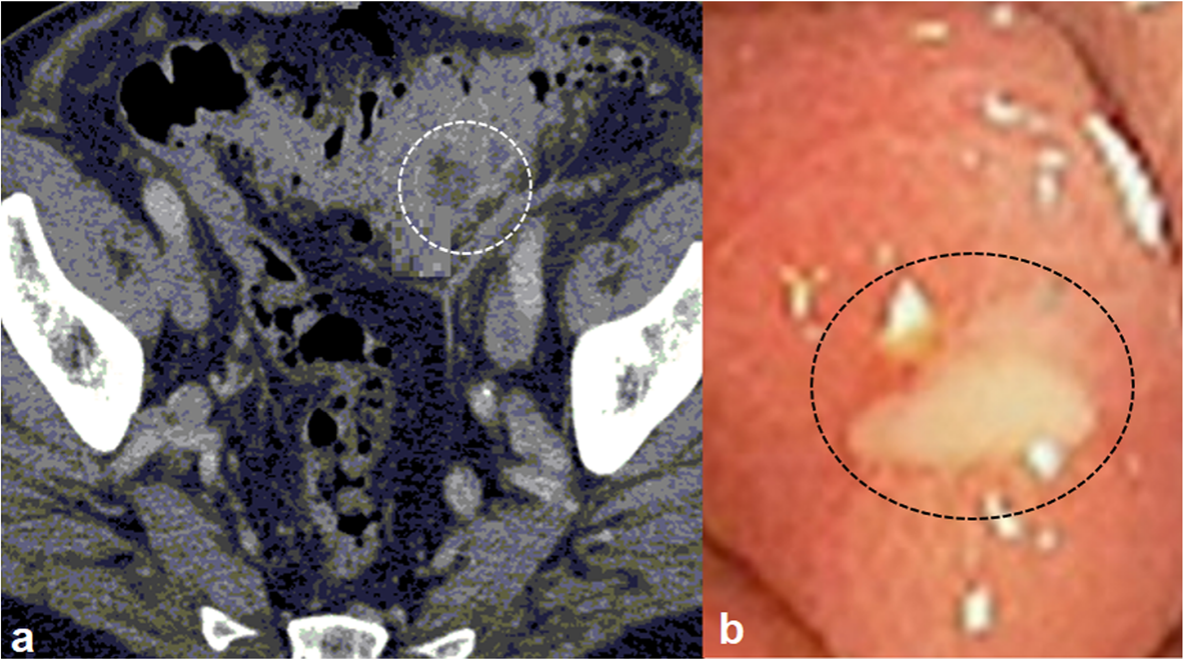
Lesion ulceration of male patients aged 51 years. **a**: Computed tomographic colonography, the white circle indicates lesion ulceration. **b**. Colonoscopy, the black circle indicates lesion ulceration

**Fig. 3:**
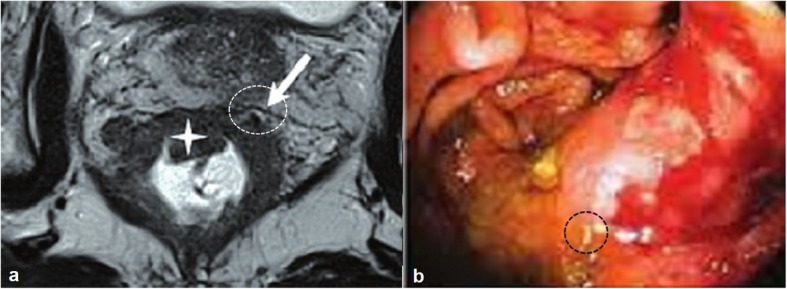
52 years old female patient with colonic extramural invasion. **a**: Computed tomographic colonography, the white circle indicates colonic extramural invasion. **b**. Colonoscopy, black circle indicates colonic extramural invasion

**Fig. 4:**
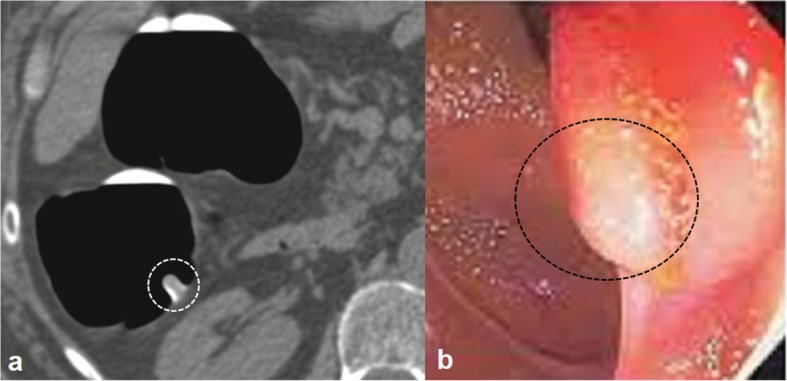
Colon lesion shouldering of 53 years old female. **a**: Computed tomographic colonography, the white circle indicates lesion shouldering. **b**. Colonoscopy, the black circle indicates lesion shouldering

Colonoscopy performed after computed tomographic colonography. Lesion ulceration (Fig. 2), extramural invasion (Fig. 3), and/ or lesion shouldering (Fig. 4) was considered as a suspicious polyp (as directed by the internal-institutional guideline for colorectal cancer). Image analyses performed by radiologists (minimum of 7-years of experience in abdominal imaging) of institutes.

### Surgery

Patients who had polyp 10 mm ø or more and polyps less than 10 mm ø but suspicious in either of modality were subjected to endoscopy (performed by endoscopists, a minimum 3-years of experience of institutes) following biopsies (performed by pathologists, a minimum 3-years of experience of institutes). Histologically (performed by pathologists, a minimum 3-years of experience of institutes, after the computed tomographic colonography and colonoscopy) confirmed suspicious polyps and 10 mm ø or more polyps (due to symptoms) were removed by colorectal surgeons (minimum of 3-years of experience) of institutes.

### Benefit score analysis

Benefit score analysis for each diagnosis modality for detection of 10 mm and more ø polyps was calculated as per Eq. 1 and that for less than 10 mm ø but suspicious polyps were calculated a were calculated as per Eq. 2 [14]:

1 $$ \mathrm{Benefit}\ \mathrm{score}=\frac{\mathrm{True}\ \mathrm{positive}\ge 10\ \mathrm{mm}\o \mathrm{polyp}}{\mathrm{Total}\ \mathrm{numbers}\ \mathrm{of}\ \mathrm{patients}\ \mathrm{diagnosed}}-\left(\frac{\mathrm{False}-\mathrm{positive}\ge 10\ \mathrm{mm}\o \mathrm{polyp}}{\mathrm{Total}\ \mathrm{numbers}\ \mathrm{of}\ \mathrm{patients}\ \mathrm{diagnosed}}\times \frac{\mathrm{Level}\ \mathrm{of}\ \mathrm{diagnostic}\ \mathrm{confidence}\ \mathrm{above}\ \mathrm{which}\ \mathrm{decision}\ \mathrm{of}\ \mathrm{surgery}\ \mathrm{was}\ \mathrm{taken}}{1-\mathrm{Level}\ \mathrm{of}\ \mathrm{diagnostic}\ \mathrm{confidence}\ \mathrm{above}\ \mathrm{which}\ \mathrm{decision}\ \mathrm{of}\ \mathrm{surgery}\ \mathrm{was}\ \mathrm{taken}}\right) $$

2 $$ \mathrm{Benefit}\ \mathrm{score}=\frac{\mathrm{True}\ \mathrm{positive}<10\ \mathrm{mm}\o \mathrm{but}\ \mathrm{the}\ \mathrm{suspicious}\ \mathrm{polyp}}{\mathrm{Total}\ \mathrm{numbers}\ \mathrm{of}\ \mathrm{patients}\ \mathrm{diagnosed}}-\left(\frac{\mathrm{False}-\mathrm{positive}<10\ \mathrm{mm}\o \mathrm{but}\ \mathrm{the}\ \mathrm{suspicious}\ \mathrm{polyp}}{\mathrm{Total}\ \mathrm{numbers}\ \mathrm{of}\ \mathrm{patients}\ \mathrm{diagnosed}}\times \frac{\mathrm{Level}\ \mathrm{of}\ \mathrm{diagnostic}\ \mathrm{confidence}\ \mathrm{above}\ \mathrm{which}\ \mathrm{decision}\ \mathrm{of}\ \mathrm{surgery}\ \mathrm{was}\ \mathrm{taken}}{1-\mathrm{Level}\ \mathrm{of}\ \mathrm{diagnostic}\ \mathrm{confidence}\ \mathrm{above}\ \mathrm{which}\ \mathrm{decision}\ \mathrm{of}\ \mathrm{surgery}\ \mathrm{was}\ \mathrm{taken}}\right) $$

### Cost

The cost of diagnosis of colorectal cancer with computed tomographic colonography (is not of diagnostic colonography and colonoscopy to confirm the positive result of computed tomographic colonography prior to surgery) and colonography was calculated.

### Adverse events

Any reported adverse effects after diagnosis procedures including hospitalization were noted.

### Statistical analysis

InStat, GraphPad, San Diego, CA, USA was used for statistical analyses. The sample size was calculated at 80% of power and a 5% level of confidence. The Fischer exact test was performed for categorical data [4]. Mann-Whitney *U*-test was used for continuous data [3]. The results were considered significant at a 95% level of confidence.

## Results

### Demographic parameters

The average age of patients was 65.12 ± 7.28 years and 183 (58%) patients were women. The enrolled patients had symptoms like abdominal pain, rectal bleeding, and/ or change in bowel habits. Patients reported one or more than one symptom suggestive of colorectal cancer and needed whole-colon examinations (Table 1). All patients had performed radiological diagnosis within 2-weeks after recommendation by the respective consultant(s).

**Table 1:** Demographic parameters and clinical conditions of diagnosed patients

Parameters		Value
Patients		318
Age (years)	Minimum	30
	Maximum	75
	Mean ± SD	65.12 ± 7.28
Gender	Male	135(42)
	Female	183(58)
Ethnicity	Han Chinese	292(92)
	Mongolian	23(7)
	Tibetan	3(1)
Symptoms	Abdominal pain	201(63)
	Rectal bleeding	53(17)
	Change in bowel habit	278(87)
	Anemia	171(54)
	Weight loss	151(48)
Route of referral	Gastroenterology	205(64)
	Colorectal surgical clinic	85(27)
	Geriatrics	28(9)

Continuous data are presented as mean ± SD and categorical data are presented as frequency (percentage)

### Diagnostic performance

Either diagnosis showed polyps 10 mm ø and more in 27 patients and polyps of 50 patients were less than 10 mm ø but suspicious. Therefore, a total of 77 patients were subjected to surgery and surgical pathology (Table 2).

**Table 2:** Performance of diagnostic methods

Parameters	Surgical pathology	Computed tomographic colonography		Colonoscopy	
Patients	77	318	^*^p-value	318	^*^p-value
Polyp ≥10 mm ø	25(32)	27(8)	0.863	23(7)	0.859
Polyps < 10 mm ø but suspicious	49(64)	47(15)		41(13)	
Non suspicious and < 10 mm ø polyps	3(4)	239(75) ^**^	< 0.0001	247(78)^**^	< 0.0001
Inconclusive results	0(0)	5(2)	0.588	7(2)	0.354

Data are presented as frequency (percentage)

^*^Respect to surgical pathology

Lesion ulceration, extramural invasion, and/ or lesion shouldering was considered as suspicious polyp

The Fischer exact test was used statistical analysis

A *p* < 0.05 was considered significant

^**^Significant difference with respect to surgical pathology

ø: Diameter

With respect to surgical pathology, sensitivities for computed tomographic colonography and colonoscopy were 0.961 and 0.831 and accuracies were reported in Table 3.

**Table 3:** Accuracies of diagnostic methods

Parameters	Computed tomographic colonography	Colonoscopy
For polyp ≥10 mm ø	0.926	0.920
For polyps < 10 mm ø but suspicious	0.959	0.837

Lesion ulceration, extramural invasion, and/ or lesion shouldering was considered as suspicious polyp

ø: Diameter

### Benefit score analysis

**Fig. 5:**
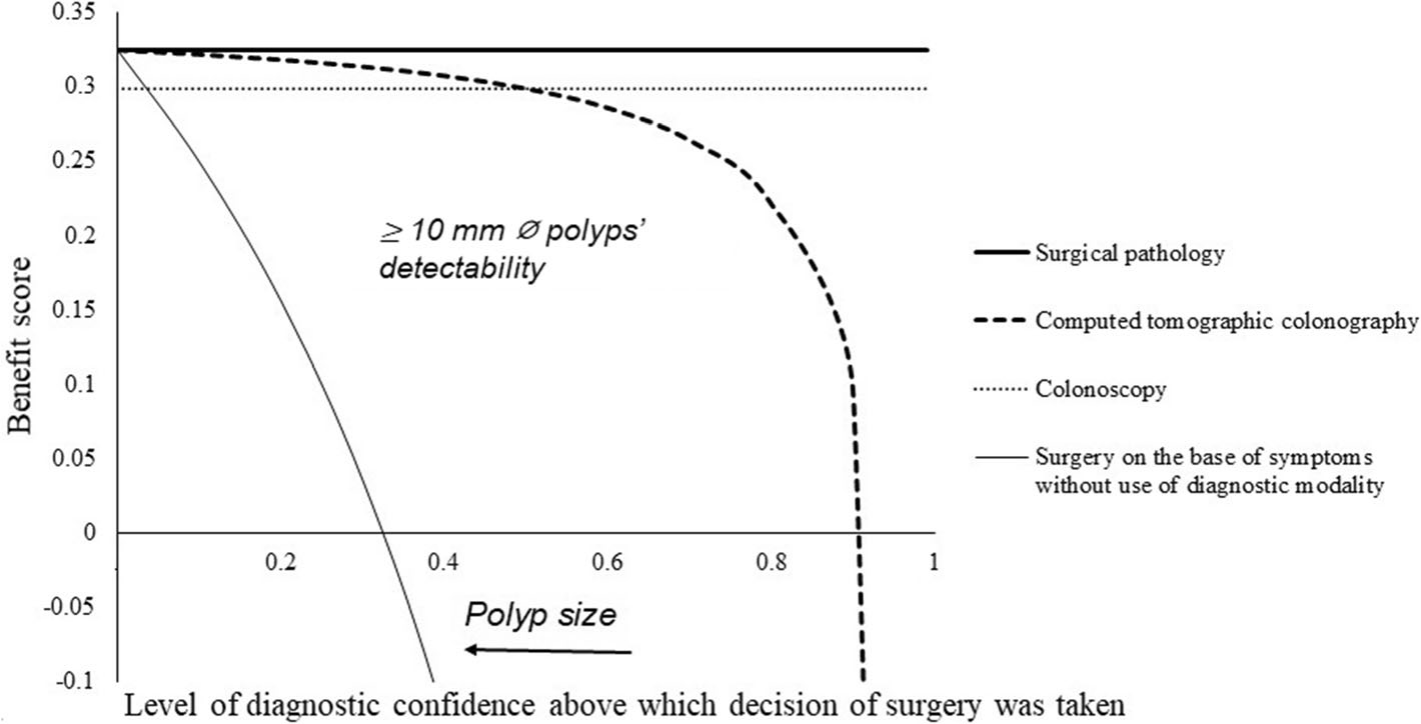
Beneficial score analysis for the diagnosis of 10 mm in diameter or more sized polyps. ø: Diameter

**Fig. 6:**
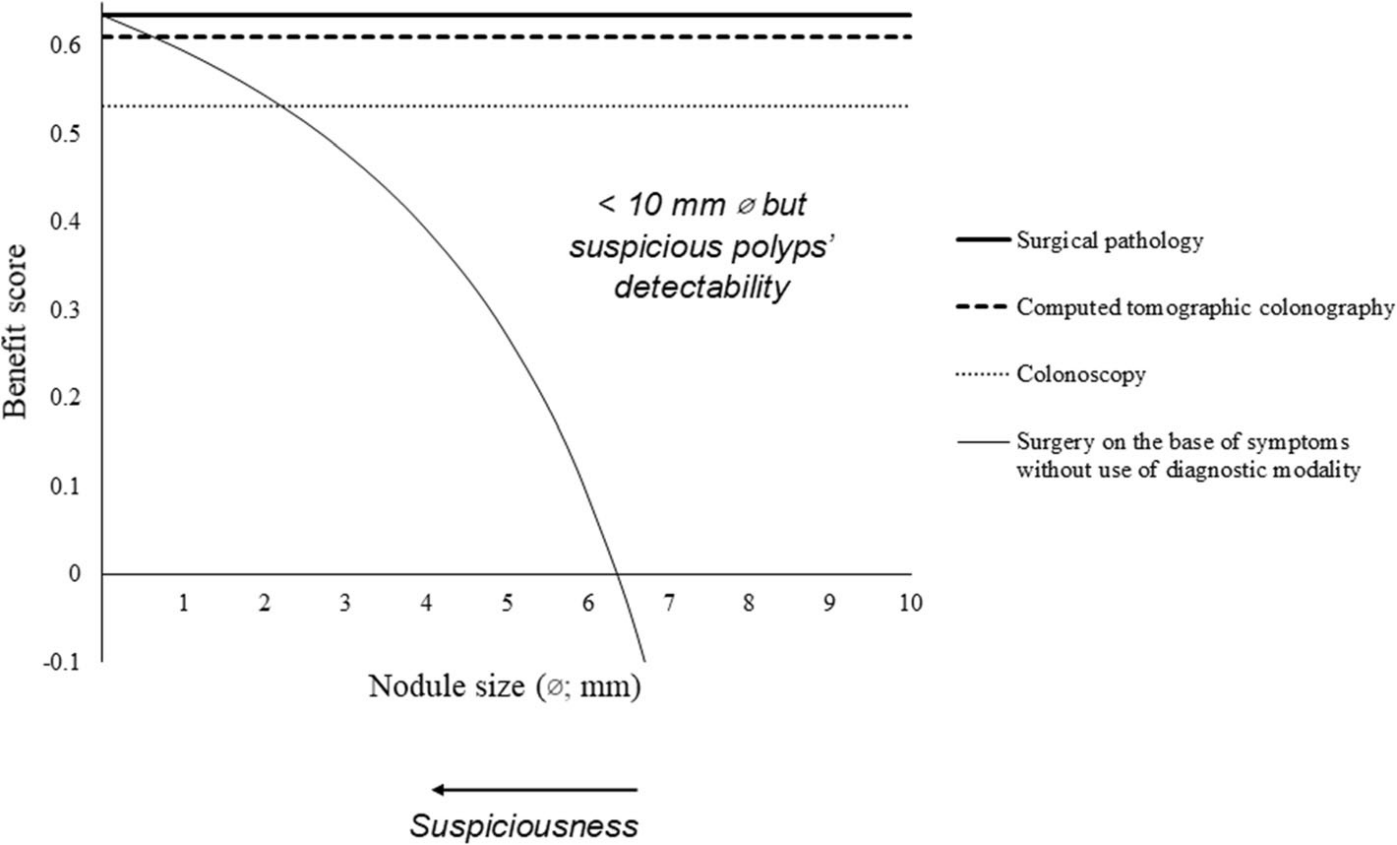
Benefit score analysis for less than 10 mm in diameter but suspicious polyps. Lesion ulceration, extramural invasion, and/ or lesion shouldering was considered as a suspicious polyp. ø: Diameter

For detection of 10 mm ø or more polyp, benefit score for computed tomographic colonography and colonoscopy were 0–0.906 diagnostic confidence and 0.035–0.5 diagnostic confidence. For polyps 10 mm ø or more but not too many large polyps, colonoscopy had a risk of underdiagnosis (Fig. 5). For less than 10 mm ø but suspicious polyps, computed tomographic colonography could detect 0.6–9.99 mm ø polyps while colonoscopy could detect 2.2–9.99 mm ø polyps. Less than 0.6 mm ø and less than 2.2 mm ø suspicious polyps could not be detected by computed tomographic colonography and colonoscopy, respectively (Fig. 6).

### Cost

**Fig. 7:**
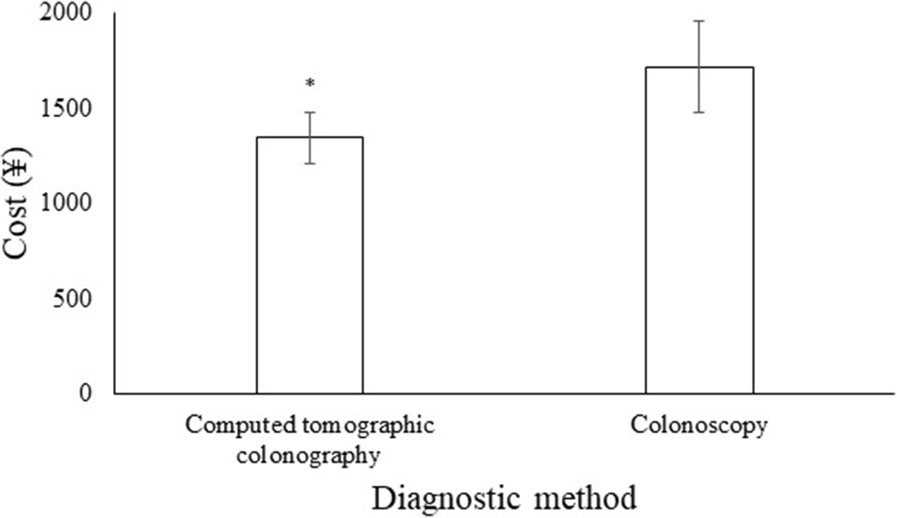
Cost-analysis of diagnosis methods for colorectal cancer diagnosis. Data are presented as mean ± SD, 318 patients were diagnosed for both modalities. Mann-Whitney *U*-test used for analysis. A *p* < 0.05 was considered significant. ^*^Significant lower cost than colonoscopy

The computed tomographic colonography had less cost than colonoscopy (1345 ± 135 ¥/ patient vs. 1715 ± 241 ¥/ patient, *p* < 0.0001) for diagnosis of colorectal cancer (Fig. 7).

### Adverse events

An unplanned hospital admission within a month occurred in two patients due to bowel perforation. No death was reported within 3-months after diagnosis.

## Discussion

The computed tomographic colonography had high sensitivity for colorectal polyps, high accuracy for polyps ≥10 mm ø and polyps < 10 mm ø but suspicious polyps, and less cost than colonoscopy. The results of the study were consistent with the SIGGAR trial [3, 4]. The computed tomographic colonography would be an alternative method than colonoscopy for detection of colorectal cancer.

The study reported that colonoscopy had underdiagnosis and computed tomographic colonography had overdiagnosis for polyps ≥10 mm ø. The results of the study were in line with the SIGGAR trial [3, 5, 15]. The different morphological characteristics of lesions and inadequate bowel preparation reduce the sensitivity of colonoscopy [16]. Extracolonic abnormalities detected by computed tomographic colonography would lead to overdiagnosis of colorectal cancer [17]. Endoscopy and contrast agents may overcome the overdiagnosis of computed tomographic colonography.

The cut-off for suspicious polyps for computed tomographic colonography was 0.6 mm ø and that for colonoscopy was 2.2 mm ø. The results of the study agreed with the statement of the second ESGAR consensus [18]. The use of endoscopy and contrast agents with computed tomographic colonography and inadequate bowel preparation were responsible for these results [4]. Evidence-based guidelines are needed for the threshold value of suspicious polyps in colorectal cancer.

Computed tomographic colonography was cost-effective than colonoscopy for a diagnosis of colorectal cancer. The results of the study were consistent with the SIGGAR trial [3] and a randomized controlled trial [19]. Biopsies and cytopathology would lead to an increase in the cost of colonoscopy [20]. The cost for diagnosis of colorectal polyps in follow-up after curative surgery is also required to consider to reach any conclusion.

Two patients reported bowel perforation and hospitalization after diagnosis. Colonoscopy is responsible for the perforation of the bowel and unplanned hospitalization [5]. The results of the study were consistent with the SIGGAR trial [3]. Computed tomographic colonography would be safe than colonoscopy.

Several limitations of the study reported, for example, retrospective reviews and lack of randomized trial but in diagnostic performance study, randomized trials are difficult to design. The extracolonic abnormalities detected by computed tomographic colonography did not report and discuss. The methods adopted for diagnosis after curative surgeries and costs for the same were not reported. In the long-term, follow-up acceptability and psychologic consequences of patients for colonoscopy are higher than the computed tomographic colonography [15] because of lifetime burden of the computed tomographic colonography [11] but data regarding acceptability, psychologic consequences, and inter-and interobserver’ agreements of the diagnosis methods did not discuss.

## Conclusions

Computed tomographic colonography had high sensitivity, high accuracy (for polyps ≥10 mm in diameter and polyps < 10 mm in diameter but suspicious polyps), less cost, and safe than colonoscopy for a diagnosis of colorectal cancer in symptomatic patients. Computed tomographic colonography had 0.6 mm in diameter and colonoscopy had a 2.2 mm in diameter threshold for suspicious polyps. Computed tomographic colonography would be a non-inferior alternative than colonoscopy for the diagnosis of colorectal cancer. A large randomized trial is required including postoperative follow-up data for diagnosis of colorectal cancer to state hypothesis clearly. The results of the current study help to the health services regarding the detection of colorectal cancer for Medicare beneficiaries.

## Data Availability

The datasets used and analyzed during the current study and a follow-up of the patients available from the corresponding author on reasonable request.

## Abbreviations

STROBE: Strengthening the reporting of observational studies in epidemiology
⌀: Diameter

## References

[1] Gu MJ, Huang QC, Bao CZ, Li YJ, Li XQ, Ye D, Ye ZH, Chen K, Wang JB (2018). Attributable causes of colorectal cancer in China. BMC Cancer.

[2] Bernstein AM, Song M, Zhang X, Pan A, Wang M, Fuchs CS, Le N, Chan AT, Willett WC, Ogino S, Giovannucci EL, Wu K (2015). Processed and unprocessed red meat and risk of colorectal cancer: analysis by tumor location and modification by time. PLoS One.

[3] Halligan S, Dadswell E, Wooldrage K, Wardle J, von Wagner C, Lilford R, Yao GL, Zhu S, Atkin W (2015). Computed tomographic colonography compared with colonoscopy or barium enema for diagnosis of colorectal cancer in older symptomatic patients: two multicentre randomised trials with economic evaluation (the SIGGAR trials). Health Technol Assess.

[4] Atkin W, Dadswell E, Wooldrage K, Kralj-Hans I, von Wagner C, Edwards R, Yao G, Kay C, Burling D, Faiz O, Teare J, Lilford RJ, Morton D, Wardle J, Halligan S, SIGGAR investigators (2013). Computed tomographic colonography versus colonoscopy for investigation of patients with symptoms suggestive of colorectal cancer (SIGGAR): a multicentre randomised trial. Lancet.

[5] Wang L, Mannalithara A, Singh G, Ladabaum U (2018). Low rates of gastrointestinal and non-gastrointestinal complications for screening or surveillance colonoscopies in a population-based study. Gastroenterology.

[6] Lovett P, Gomez V, Hodge DO, Ladlie B (2017). Propofol versus midazolam/fentanyl sedation for colonoscopy in the elderly patient population. J Perianesth Nurs.

[7] Meiklejohn DJ, Ridley LJ, Ngu MC, Cowlishaw JL, Duller A, Ridley W (2018). Utility of minimal preparation computed tomography colonography in detecting colorectal cancer in elderly and frail patients. Intern Med J.

[8] Pooler BD, Lubner MG, Theis JR, Halberg RB, Liang Z, Pickhardt PJ (2019). Volumetric textural analysis of colorectal masses at CT colonography: differentiating benign versus malignant pathology and comparison with human reader performance. Acad Radiol.

[9] Pickhardt PJ, Correale L, Delsanto S, Regge D, Hassan C (2018). CT colonography performance for the detection of polyps and cancer in adults ≥ 65 years old: systematic review and meta-analysis. Am J Roentgenol.

[10] Yee J, McFarland E (2018). Extracolonic findings and radiation at CT colonography: what the referring provider needs to know. Abdom Radiol.

[11] Knudsen AB, Zauber AG, Rutter CM, Naber SK, Doria-Rose VP, Pabiniak C, Johanson C, Fischer SE, Lansdorp-Vogelaar I, Kuntz KM (2016). Estimation of benefits, burden, and harms of colorectal cancer screening strategies: modeling study for the us preventive services task force. JAMA.

[12] Halligan S, Wooldrage K, Dadswell E, Kralj-Hans I, von Wagner C, Edwards R, Yao G, Kay C, Burling D, Faiz O, Teare J, Lilford RJ, Morton D, Wardle J, Atkin W, SIGGAR investigators (2013). Computed tomographic colonography versus barium enema for diagnosis of colorectal cancer or large polyps in symptomatic patients (SIGGAR): a multicentre randomised trial. Lancet.

[13] Columb MO, Atkinson MS (2015). Statistical analysis: sample size and power estimations. BJA Educ.

[14] Li J, Yu Y, Zhu L, Li Y, He Q (2020). Magnetic resonance imaging versus computed tomography for biliary tract intraductal papillary mucinous neoplasm (BT-IPMN): A diagnostic performance analysis. Med Sci Monit.

[15] von Wagner C, Ghanouni A, Halligan S, Smith S, Dadswell E, Lilford RJ, Morton D, Atkin W, Wardle J, Investigators SIGGAR (2012). Patient acceptability and psychologic consequences of CT colonography compared with those of colonoscopy: results from a multicenter randomized controlled trial of symptomatic patients. Radiology.

[16] Rabeneck L, Paszat LF (2010). Circumstances in which colonoscopy misses cancer. Frontline Gastroenterol.

[17] Halligan S, Wooldrage K, Dadswell E, Shah U, Kralj-Hans I, von Wagner C, Faiz O, Teare J, Edwards R, Kay C, Yao G, Lilford RJ, Morton D, Wardle J, Atkin W, Investigators SIGGAR (2015). Identification of extracolonic pathologies by computed tomographic colonography in colorectal cancer symptomatic patients. Gastroenterology.

[18] Neri E, Halligan S, Hellstrom M, Lefere P, Mang T, Regge D, Stoker J, Taylor S, Laghi A, ESGAR CT Colonography Working Group (2013). The second ESGAR consensus statement on CT colonography. Eur Radiol.

[19] van der Meulen MP, Lansdorp-Vogelaar I, Goede SL, Kuipers EJ, Dekker E, Stoker J, van Ballegooijen M (2018). Colorectal cancer: cost-effectiveness of colonoscopy versus CT colonography screening with participation rates and costs. Radiology.

[20] Hassan C, Pickhardt PJ (2013). Cost-effectiveness of CT colonography. Radiol Clin N Am.

